# Mediterranean Diet Adherence and Sustainable Eating Behaviors in Type 2 Diabetes: A Cross-Sectional Analysis of Adiposity, Cooking and Food Skills, and Macrovascular Complications

**DOI:** 10.3390/healthcare14182934

**Published:** 2026-09-09

**Authors:** Hatice Ozcaliskan Ilkay, Zuleyha Cihan Ozdamar Karaca

**Affiliations:** 1 Department of Nutrition and Dietetics, Faculty of Health Sciences, Erciyes University, 38260 Kayseri, Türkiye; 2 Department of Endocrinology, Faculty of Medicine, Erciyes University, 38030 Kayseri, Türkiye

**Keywords:** sustainable eating, food skills, diet, obesity, vascular health, type 2 diabetes mellitus

## Abstract

**Highlights:**

**What are the main findings?**

- Greater Mediterranean diet adherence was cross-sectionally associated with more sustainable and healthy eating behaviors.
- Higher sustainable eating behavior scores were associated with a lower waist-to-hip ratio after correction for multiple testing.

**What are the implications of the main findings?**

- Mediterranean diet adherence and sustainable eating behaviors may represent relevant targets for future dietary interventions in adults with type 2 diabetes.
- Prospective and intervention studies are needed to determine whether modifying these dietary behaviors improves subsequent cardiometabolic and vascular outcomes.

**Abstract:**

**Aims:** Although the Mediterranean diet (MD) is widely recognized as a sustainability-aligned dietary model, its relationship with sustainable eating behaviors and its cross-sectional associations with adiposity, cooking and food skills, and macrovascular complications in type 2 diabetes mellitus (T2DM) remain insufficiently characterized. **Methods:** This cross-sectional study included 120 adults with T2DM (median age, 60 years) consecutively recruited from an endocrinology outpatient clinic. Mediterranean diet adherence (MDA), Sustainable and Healthy Eating Behaviors (SHEB), and Cooking Skills and Food Skills (CSFS) were assessed using validated scales. Dietary intake was evaluated using a single 24-h dietary recall, and anthropometric and body composition measures were obtained using standardized procedures. Adjusted group differences were examined using ANCOVA. To account for multiplicity across prespecified families of exploratory outcomes, nominal *p*-values were corrected using the Benjamini–Hochberg false discovery rate (FDR) procedure. **Results:** Higher MDA was associated with a higher total SHEB score (primary outcome; *p* < 0.001) and with higher scores for quality labels, seasonal foods and food waste avoidance, local food, and reduced meat consumption, after FDR correction (all q < 0.05). Across SHEB tertiles, waist-to-hip ratio differed significantly after FDR correction (*p* = 0.001, q = 0.010), whereas the nominal difference in neck circumference did not remain significant (*p* = 0.046, q = 0.230). In analyses according to macrovascular complication status, lower adjusted protein intake remained significant after FDR correction (*p* = 0.002, q = 0.032). Nominal differences in food skills, total CSFS, MDA, total and saturated fat intake, monounsaturated fat intake, and dietary total antioxidant capacity did not remain significant after multiplicity correction (all q > 0.05). **Conclusions:** Greater adherence to the Mediterranean diet was cross-sectionally associated with more sustainable and healthier eating behaviors, whereas higher SHEB scores were associated with a lower waist-to-hip ratio. Most exploratory differences according to macrovascular complication status did not remain significant after correction for multiple testing, underscoring the need for cautious interpretation. Prospective studies are needed to establish temporal relationships, and intervention studies are required to determine whether modifying adherence to the Mediterranean diet, sustainable eating behaviors, or cooking and food skills affect subsequent cardiometabolic and vascular outcomes in T2DM.

## 1. Introduction

Dietary patterns that integrate human health, environmental sustainability, and long-term disease management have gained increasing attention in the context of type 2 diabetes mellitus (T2DM), where nutritional strategies must address both metabolic control and cardiovascular risk [1,2]. Among dietary patterns addressing these multidimensional demands, the Mediterranean diet (MD) stands out as a well-established model that integrates cardiometabolic health with environmental sustainability and cultural food traditions [3]. It is characterized by a high intake of plant-based foods—such as whole grains, fruits, vegetables, legumes, and nuts—moderate consumption of fish, white meat, eggs, and dairy products, limited intake of red and processed meats, and olive oil as the primary source of dietary fat [4]. Beyond its nutritional value, the MD supports biodiversity by encouraging the consumption of locally produced, seasonal foods and is increasingly recognized for its relatively low environmental footprint. Given the substantial environmental impact of food systems, a shift toward sustainable dietary patterns such as the Mediterranean diet may foster environmentally responsible and health-promoting eating behaviors [3,5].

Although numerous dietary approaches with strict prescriptions for individuals with type 2 diabetes have demonstrated short-term benefits in reducing HbA1c levels and/or inducing weight loss, the long-term sustainability of these effects remains challenging [6,7,8]. Weight regain after discontinuation of the prescribed diet and reversal of improvements in glycemic control are common outcomes observed in both research settings and routine clinical practice [7]. In this context, growing evidence suggests that sustainable and health-oriented eating behaviors may offer a more durable alternative by promoting balanced, plant-forward, and environmentally conscious food choices while fostering long-term diabetes self-management [9,10,11]. Diets aligned with sustainability principles are typically rich in fiber, antioxidants, and bioactive compounds, which enhance satiety, support metabolic regulation, and reduce systemic inflammation [12]. Accordingly, population-based studies have consistently shown that greater adherence to sustainable dietary patterns and more positive attitudes toward sustainable eating are associated with lower body weight and more favorable anthropometric profiles [13,14]. Collectively, these properties may contribute to improved vascular health and a lower risk of atherosclerotic and macrovascular complications [4,15].

While sustainable dietary patterns have been associated with favorable metabolic and cardiovascular profiles, translating these principles into daily practice remains challenging for many individuals with T2DM [2,16]. Strengthening practical cooking and food skills may facilitate the adoption and maintenance of realistic and sustainable dietary practices [17,18]. Previous intervention studies have also suggested potential benefits of skill-based dietary approaches for diabetes self-management and glycemic outcomes [19]; however, their effects on subsequent cardiovascular events remain to be established.

Dietary patterns combining favorable nutrient composition with sustainability principles—most notably the Mediterranean diet—have been associated with cardiovascular outcomes in individuals with T2DM [15]. Within this framework, accumulating evidence suggests that the quality of such sustainable, cardioprotective dietary patterns may modulate macrovascular risk through interrelated mechanisms involving atherogenic dyslipidemia, endothelial dysfunction, oxidative stress, and chronic low-grade inflammation [4]. In this context, both the amount and source of dietary protein, as well as the quantity and quality of dietary fat, have been implicated in cardiometabolic pathways relevant to vascular health, with particular emphasis on reducing saturated fat intake and favoring unsaturated fatty acids to support lipid metabolism and glycemic regulation [20,21,22,23]. In addition, antioxidant-rich dietary profiles may further contribute to vascular protection by attenuating oxidative stress and inflammatory processes central to atherosclerosis [24,25].

The primary objective of this study was to examine the association between Mediterranean diet adherence and overall sustainable and healthy eating behavior in adults with type 2 diabetes mellitus. For the purposes of the present analysis, the total SHEB score was designated as the primary outcome, while analyses of individual SHEB dimensions were considered exploratory and used to further characterize the overall association. Additional exploratory analyses examined differences in anthropometric and body composition measures across levels of sustainable eating behavior, and differences in cooking and food skills, Mediterranean diet adherence, and selected dietary characteristics according to macrovascular complication status.

## 2. Materials and Methods

### 2.1. Study Design and Data Collection

The present cross-sectional study included 120 adults with T2DM who were consecutively recruited from the Endocrinology and Metabolism Outpatient Clinic of Erciyes University Hospital between November 2024 and March 2025. Participants were 18–75 years of age, had been clinically diagnosed with T2DM for at least one year, and had no changes in their medical treatment within the previous three months. Exclusion criteria included pregnancy or lactation, cognitive impairment, autoimmune disease, systemic infection, terminal illness, malignancy, and regular use of antibiotics or probiotics during the preceding three months. Trained intern dietitians conducted face-to-face interviews using a structured questionnaire with participants who met the inclusion criteria and provided informed consent. The questionnaire included sections on sociodemographic and descriptive characteristics, anthropometric measurements, biochemical findings, and blood pressure levels. The presence of diabetes-related chronic complications (macrovascular and microvascular) was determined during routine clinical evaluations by physicians at the Endocrinology and Metabolism Outpatient Clinic and/or verified from participants’ medical records. For the present analyses, macrovascular complication status was classified as the absence versus the presence of at least one documented macrovascular complication. This classification was based on routinely documented clinical information and should not be interpreted as a formally adjudicated cardiovascular composite endpoint. Individual component diagnoses were not systematically coded separately; however, a history of myocardial infarction and stroke was separately available for 5 and 3 participants, respectively. Dietary habits, food consumption frequency, and a single 24-h dietary recall were administered. Furthermore, several validated instruments were incorporated to evaluate sustainable nutrition behaviors and related factors, including the Sustainable and Healthy Eating Behaviors Scale, the Cooking Skills and Food Skills Scale, and the Mediterranean Diet Adherence Scale. Physical activity levels were assessed using the International Physical Activity Questionnaire–Short Form (IPAQ-SF).

The sample size was determined a priori within the framework of the broader research project from which the present data were derived. The original sample size calculation, performed using G*Power version 3.1, indicated a minimum requirement of 100 participants (f = 0.35, power = 80%, α = 0.05), and 120 participants were ultimately recruited to account for potential data loss. This sample-size calculation was performed for the broader parent project and was not specifically based on the primary outcome, subgroup comparisons, or adjusted models examined in the present analysis; therefore, it should not be interpreted as an a priori power calculation for all analyses reported herein.

This study was approved by the Erciyes University Health Sciences Research Ethics Committee (approval date: 7 August 2024; approval/decision no.:2024/112, application no.: 96681246) and was performed in line with the principles of the Declaration of Helsinki. Written informed consent was obtained from all participants before data collection.

### 2.2. Sustainable and Healthy Eating Behaviors (SHEB) Scale

The Sustainable and Healthy Eating Behaviors (SHEB) Scale was originally developed by Żakowska-Biemans et al. [26] to assess how the concept of sustainable and healthy eating is interpreted among the adult population and to measure self-reported sustainable and healthy eating behaviors. The Turkish validity and reliability study of the scale was conducted by Köksal et al. [27]. The scale consists of 32 items, covering the following dimensions: healthy and balanced diet, quality labels (regional and organic), reduction in meat consumption, local food, low-fat foods, seasonal food, avoiding food waste, and animal welfare. Responses are rated on a seven-point Likert scale, ranging from 1 (never) to 7 (always).

In the present sample, internal consistency was good for the overall SHEB scale (Cronbach’s α = 0.858). Internal-consistency estimates for the subdimensions, in the same order, were α = 0.812 for healthy and balanced diet (4 items), α = 0.741 for quality labels (8 items), α = 0.705 for reduction in meat consumption (3 items), α = 0.741 for local food (3 items), α = 0.615 for low-fat foods (3 items), α = 0.597 for seasonal food and avoiding food waste (7 items), and α = 0.304 for animal welfare (4 items). The original validated subscale structure was retained without post hoc item deletion.

### 2.3. Cooking Skills and Food Skills (CSFS) Scale

The Cooking Skills and Food Skills (CSFS) Scale was developed by Lavelle et al. [28] to measure individuals’ self-perceived cooking and food preparation abilities. The Turkish adaptation and validation of the scale were performed by Keleş et al. [29]. The scale consists of 33 items grouped under two subscales: cooking skills (14 items) and food preparation skills (19 items). The scale is rated on a 7-point Likert scale, ranging from 1 = very poor to 7 = very good, with an additional option of never/rarely. Participants who selected the “never/rarely” option received a score of 0. Higher scores indicate a higher level of perceived proficiency in cooking and food preparation.

In the present sample, internal consistency was high for the CSFS total score (Cronbach’s α = 0.913; 33 items) and for the Cooking Skills (α = 0.896; 14 items) and Food Skills (α = 0.838; 19 items) subscales.

### 2.4. Mediterranean Diet Adherence (MDA) Scale

Adherence to the Mediterranean diet was assessed using the 14-item Mediterranean Diet Adherence (MDA) Scale originally developed by Martínez-González et al. [30] and subsequently validated for the Turkish population by Bekar et al. [31]. The MDA scale comprises two items on general food intake habits and twelve items on the frequency of consumption of specific food groups characteristic of the Mediterranean diet. Each item is scored 0 or 1, yielding a total score ranging from 0 to 14. Higher scores indicate greater adherence to the Mediterranean dietary pattern, whereas lower scores reflect poorer adherence. Based on established MEDAS classifications, participants were categorized as having low (≤5 points), moderate (6–9 points), or high (≥10 points) adherence [32,33].

### 2.5. Dietary Intake Assessment and Nutrient Analysis

Dietary intake was assessed using a single 24-h dietary recall administered by trained dietitians. Participants were asked to report all foods and beverages consumed during the preceding 24 h. Portion sizes and amounts consumed were estimated using the Food and Meal Photograph Catalog: Measurements and Quantities (Yemek ve Besin Fotoğraf Kataloğu: Ölçü ve Miktarlar) by Rakıcıoğlu et al. [34]. Reported foods and beverages were subsequently entered into the BeBiS (Beslenme Bilgi Sistemi, version 9.0; University of Hohenheim, Stuttgart, Germany) dietary analysis software, which was used to calculate daily energy and nutrient intakes. ORAC values and dietary total antioxidant capacity were obtained directly from the corresponding outputs generated by BeBiS; no separate external antioxidant database was used by the investigators.

### 2.6. International Physical Activity Questionnaire (IPAQ)-Short Form

Physical activity was evaluated using the International Physical Activity Questionnaire (IPAQ), a widely applied self-report tool [35]. The Turkish version, validated by Sağlam et al. [36], has demonstrated strong reliability and validity among Turkish adults. The IPAQ assesses physical activity performed during the previous seven days across multiple domains, including work, transportation, household, and leisure-time activities. Physical activity levels are expressed in MET-minutes per week, and participants are classified as having low (<600 MET-min/week), moderate (600–2999 MET-min/week), or high (≥3000 MET-min/week) physical activity, in accordance with international IPAQ scoring protocols.

### 2.7. Anthropometric and Body Composition Measurements

Anthropometric data—including height, body weight, waist circumference (WC), hip circumference, and neck circumference—were collected once by trained dietetic interns following standardized procedures [37,38]. Participants were measured barefoot and wearing light clothing, and were instructed to breathe normally during the assessments. Body weight was recorded to the nearest 0.1 kg using a digital scale (Seca 803; Seca GmbH & Co. KG, Hamburg, Germany), while height was measured to the nearest 0.1 cm with a stadiometer (MZ10032; Tanita Corporation, Tokyo, Japan). Waist and hip circumferences were measured using a non-elastic tape (Seca GmbH & Co. KG, Hamburg, Germany); WC was taken at the midpoint between the lowest rib and the iliac crest, and hip circumference at the widest part of the buttocks. These measurements were used to calculate body mass index (BMI; kg/m^2^) and waist-to-hip ratio (WHR) [37,38]. Neck circumference was measured with a non-stretchable tape placed just below the laryngeal prominence (Adam’s apple) and positioned perpendicular to the long axis of the neck [39]. Body composition was assessed using a portable bioelectrical impedance analyzer (TBF-410; Tanita Corporation, Tokyo, Japan) based on the tetrapolar bioelectrical impedance analysis (BIA) method. The measurements provided estimates of body fat mass (kg), body fat percentage (%), fat-free mass (kg), and total body water (kg).

### 2.8. Measurement of Plasma Metabolites and Biochemical Parameters

Venous blood samples were collected from all participants following a minimum fasting period of 8 h. Routine biochemical parameters were obtained from clinical records and analyzed using standardized laboratory procedures. Fasting plasma glucose concentrations were measured by an enzymatic spectrophotometric method (Cobas 6000 analyzer; Roche Diagnostics GmbH, Mannheim, Germany). Glycated hemoglobin (HbA1c) levels were determined using a turbidimetric inhibition immunoassay (Tina-quant HbA1c Gen.3; Roche Diagnostics GmbH, Mannheim, Germany). Serum lipid profile parameters—including total cholesterol, high-density lipoprotein cholesterol (HDL-C), low-density lipoprotein cholesterol (LDL-C), and triglycerides—were assessed using enzymatic spectrophotometric assays. All biochemical analyses were conducted at the Biochemistry Laboratory of Erciyes University Hospital and the GENKÖK Biochemistry Laboratory (Genome and Stem Cell Center, Erciyes University).

### 2.9. Statistical Analysis

Statistical analyses were conducted using IBM SPSS Statistics software, version 23.0 (Armonk, NY, USA). The normality of continuous variables was evaluated using histograms, probability plots, and the Kolmogorov–Smirnov test. In the baseline analyses, continuous variables were presented as mean ± standard deviation, with median values in parentheses, and categorical variables as frequencies and percentages. In subsequent analyses, continuous variables were reported as crude means with standard deviations or adjusted means with standard errors. Crude means represent unadjusted group averages and are provided for descriptive purposes only, whereas adjusted means were estimated using analysis of covariance (ANCOVA). ANCOVA models were adjusted for age, duration of type 2 diabetes mellitus, sex, educational level, marital status, antidiabetic medication use, insulin therapy, and total physical activity level. For analyses involving dietary variables, total energy intake was additionally included as a covariate.

Given the small number of participants in the high-MDA category, a complementary multivariable linear regression analysis was performed, modeling MDA score as a continuous variable and total SHEB score as the dependent variable, using the same covariate set as in the primary analysis. This analysis was conducted to assess the robustness of the primary association while avoiding information loss associated with categorization. Model assumptions and diagnostics were evaluated by visual inspection of residual-versus-predicted plots and normal P–P plots, together with standardized residuals, Cook’s distance, leverage values, the Durbin–Watson statistic, and variance inflation factors (VIFs). ANCOVA assumptions were assessed using appropriate model diagnostics, including the homogeneity of regression slopes, residual distributions, residual-versus-predicted plots, studentized residuals, Cook’s distance, and leverage values. Diagnostic assessments indicated that model assumptions were generally adequately met. Dietary total antioxidant capacity was a notable exception, showing marked residual non-normality and evidence of an influential observation on the original scale; therefore, a sensitivity analysis was performed using natural-log-transformed values. Detailed ANCOVA diagnostic assessments and sensitivity analyses for dietary variables showing notable departures from model assumptions are provided in Supplementary Table S1. To further evaluate the potential influence of residual vascular confounding in analyses according to macrovascular complication status, sensitivity ANCOVA models were additionally fitted, incorporating available major vascular risk factors, including smoking status, hypertension, HbA1c, LDL cholesterol, and serum creatinine, together with age, diabetes duration, sex, antidiabetic medication use, insulin therapy, physical activity, and total energy intake for dietary outcomes. These expanded models were treated as sensitivity analyses rather than replacements for the prespecified primary models due to the relatively small sample size and the increased number of covariates.

To account for multiplicity across exploratory outcomes, conceptually related analyses were grouped into three families: (1) individual Sustainable and Healthy Eating Behaviors (SHEB) subdimensions across Mediterranean Diet Adherence (MDA) categories; (2) anthropometric and body composition measures across SHEB tertiles; and (3) cooking and food skills, MDA, and dietary variables according to macrovascular complication status. Within each exploratory family, *p*-values from the overall ANCOVA tests were adjusted for multiple testing using the Benjamini–Hochberg false discovery rate (FDR) procedure. The total SHEB score, designated a priori as the primary outcome, was not included in the FDR correction applied to exploratory outcomes. For outcomes that remained significant after FDR correction, Bonferroni-adjusted pairwise post-hoc comparisons were performed where applicable. Both nominal *p*-values and FDR-adjusted q-values are reported to distinguish nominal associations from findings that remained statistically significant after correction for multiple testing. A two-sided nominal *p*-value <0.05 was used for the primary outcome, whereas an FDR-adjusted q-value < 0.05 was considered statistically significant for exploratory outcome families.

Additional exploratory analyses were conducted for fasting plasma glucose and lipid-profile parameters; however, these variables were not included among the primary or prespecified exploratory outcome families and were not subjected to the FDR correction described above.

## 3. Results

The baseline sociodemographic, clinical, and lifestyle characteristics of adults with type 2 diabetes mellitus are summarized in Table 1. A total of 120 adults with T2DM were included in the study (84.2% women; mean ± SD age, 58.6 ± 10.0 years). The median duration of diabetes was 10.5 years, and the mean BMI was 32.9 ± 6.1 kg/m^2^, with 64.2% of participants classified as obese (BMI ≥ 30 kg/m^2^). Approximately one-third of participants (31.7%, n = 38) had at least one documented macrovascular complication. Most participants were married (82.5%), had completed primary education or below (61.7%), and reported financial adequacy (63.3%). The majority were receiving oral antidiabetic therapy (93.3%), and 45.8% were also treated with insulin. The mean HbA1c level was 7.8 ± 1.7%. The median total scores for SHEB, CSFS, and MDA were 131.0, 163.5, and 6.0, respectively.

Crude and adjusted mean scores of the SHEB subdimensions and total score according to MDA categories are presented in Table 2. The total SHEB score, designated as the primary outcome, increased progressively across MDA categories, from 122.7 ± 2.7 in the low-adherence group to 146.5 ± 5.1 in the high-adherence group (*p* < 0.001, partial η^2^ = 0.175). Among the seven exploratory SHEB subdimensions, differences in quality labels (*p* = 0.001, q = 0.002), seasonal foods and food waste avoidance (*p* = 0.011, q = 0.019), local food (*p* = 0.020, q = 0.028), reduced meat consumption (*p* < 0.001, q = 0.001), and animal welfare (*p* < 0.001, q < 0.001) remained significant after FDR correction. However, the animal-welfare finding should be interpreted with particular caution because of the very low internal consistency of this subdimension in the present sample (Cronbach’s α = 0.304). Among the more reliably measured subdimensions, the largest effect size was observed for reduced meat consumption (partial η^2^ = 0.136). Bonferroni-adjusted pairwise comparisons generally indicated higher scores among participants with moderate or high MDA than among those with low adherence; however, for local food, the overall FDR-corrected association was significant, but no individual pairwise comparison remained significant after Bonferroni adjustment. Healthy and balanced eating and low-fat choices did not differ significantly across MDA categories after FDR correction. In a complementary multivariable analysis treating MDA as a continuous variable, higher MDA scores were associated with higher total SHEB scores after adjustment for the same covariates (B = 4.13, 95% CI: 2.72–5.54; standardized β = 0.493, *p* < 0.001). Each one-point increase in MDA score corresponded to an approximately 4.1-point higher total SHEB score. The overall model was statistically significant (F(9, 110) = 4.26, *p* < 0.001; adjusted R^2^ = 0.198). Model diagnostics indicated no substantial violations of regression assumptions, with no evidence of marked non-normality, heteroscedasticity, influential observations, or problematic multicollinearity (maximum Cook’s distance = 0.15; VIF range: 1.07–1.51).

Crude and adjusted mean values of anthropometric and body composition measures across tertiles of the total SHEB score are presented in Table 3. Among the 10 exploratory outcomes, waist-to-hip ratio (WHR) was the only measure that remained significantly different across SHEB tertiles after FDR correction (*p* = 0.001, q = 0.010, partial η^2^ = 0.121). Participants in the highest SHEB tertile (>140) had a lower adjusted WHR than those in the lowest tertile (0.90 ± 0.01 vs. 0.95 ± 0.01), with the pairwise difference remaining significant after Bonferroni adjustment. Neck circumference showed a nominal between-group difference (*p* = 0.046, partial η^2^ = 0.055), but this association did not remain significant after FDR correction (q = 0.230). No evidence of differences after multiplicity correction was observed for body weight, BMI, waist circumference, hip circumference, body fat percentage, fat mass, fat-free mass, or total body water (all q > 0.05).

Crude and adjusted mean values of cooking skills, food skills, CSFS total score, SHEB total score, MDA score, and dietary variables according to macrovascular complication status are presented in Table 4. After adjustment for covariates and correction for multiple testing within this exploratory family, adjusted protein intake was the only dietary outcome that remained significantly different according to macrovascular complication status (*p* = 0.002, q = 0.032, partial η^2^ = 0.088). Notably, the direction of the between-group difference in protein intake reversed after covariate and total-energy adjustment. The crude mean protein intake was numerically higher among participants with macrovascular complications than among those without complications (51.12 ± 24.39 vs. 47.42 ± 18.31 g/day), whereas the corresponding adjusted mean was lower in participants with macrovascular complications (42.60 ± 2.15 vs. 51.37 ± 1.39 g/day). Thus, the observed association reflects a difference in adjusted protein intake and should not be interpreted as indicating lower unadjusted absolute protein intake among participants with macrovascular complications.

Several additional variables showed nominal between-group differences that did not remain statistically significant after FDR correction. These included food skills (*p* = 0.010, q = 0.056), CSFS total score (*p* = 0.013, q = 0.056), MDA score (*p* = 0.018, q = 0.056), total fat (*p* = 0.046, q = 0.092), saturated fatty acids (*p* = 0.018, q = 0.056), monounsaturated fatty acids (*p* = 0.021, q = 0.056), and dietary total antioxidant capacity (*p* = 0.026, q = 0.059). No evidence of differences was observed for the remaining outcomes after multiplicity correction (all q > 0.05). Accordingly, except for protein intake, differences according to macrovascular complication status should be regarded as nominal exploratory signals rather than statistically robust findings. Dietary total antioxidant capacity showed a nominal between-group difference on the original scale (*p* = 0.026, q = 0.059); however, residual diagnostics indicated substantial non-normality. After natural-log transformation, residual diagnostics improved substantially, and the between-group difference was no longer evident (F = 0.387, *p* = 0.535), indicating that the original-scale finding was not robust to sensitivity analysis. Detailed model diagnostics are presented in Supplementary Table S1. Notably, logarithmic transformation of dietary total antioxidant capacity substantially improved the residual distribution (standardized residual skewness: 4.967 to −0.327; kurtosis: 34.125 to 0.476; Shapiro–Wilk *p* < 0.001 to *p* = 0.495) and reduced the maximum Cook’s distance from 0.78 to 0.12, while the substantive conclusion remained unchanged. In additional sensitivity analyses incorporating available major vascular risk factors, including smoking status, hypertension, HbA1c, LDL cholesterol, and serum creatinine, together with the covariates included in the primary models, the between-group difference in total protein intake remained evident (54.85 ± 2.76 vs. 45.07 ± 3.07 g/day; F = 13.326, *p* < 0.001, partial η^2^ = 0.113). Nominal between-group differences were also observed for total fat (F = 5.252, *p* = 0.024, partial η^2^ = 0.048), saturated fatty acids (F = 7.609, *p* = 0.007, partial η^2^ = 0.068), and monounsaturated fatty acids (F = 7.085, *p* = 0.009, partial η^2^ = 0.063). In contrast, no evidence of between-group differences was observed for plant protein (*p* = 0.678), polyunsaturated fatty acids (*p* = 0.309), dietary cholesterol (*p* = 0.465), or ORAC (*p* = 0.064). The log-transformed dietary total antioxidant capacity also remained unrelated to macrovascular complication status (F = 0.069, *p* = 0.793, partial η^2^ = 0.001). Full results of the expanded sensitivity analyses are provided in Supplementary Table S2. Overall, these analyses supported the robustness of the principal protein finding to additional adjustment for available vascular risk factors, whereas findings for the remaining dietary variables should be regarded as exploratory.

Additional exploratory analyses of fasting plasma glucose and lipid-profile parameters did not provide evidence of consistent associations with the study exposures.

## 4. Discussion

The Mediterranean diet encompasses all key components of sustainability—including food security and accessibility, environmental and biodiversity preservation, fair trade, local and seasonal production, and the protection of cultural heritage and traditional food skills—making it a model example of a healthy and sustainable dietary pattern [3]. Moreover, in a meta-analysis of 32 studies evaluating various dietary patterns—including the current Italian, Spanish, Western-style, Atlantic, EAT-Lancet, high-protein, vegetarian, and vegan diets—the Mediterranean diet was shown to have a lower environmental impact, higher diet quality, and comparable cost relative to other dietary patterns [40]. In this context, Metin et al. [5], in a web-based cross-sectional study among healthy adults in Türkiye, demonstrated that adherence to the Mediterranean diet was associated with significantly higher total and subscale scores on the SHEB Scale compared with non-adherence (*p* < 0.001 for all comparisons). Similarly, in an online survey of American university students, multivariate logistic regression analysis revealed that participants with higher Sustainable Healthy Diet Index scores and those who reported a stronger willingness to purchase and consume healthy and sustainable foods were more likely to adhere to the Mediterranean diet [3]. Consistent with previous studies, the present findings showed a clear cross-sectional association between Mediterranean diet adherence and sustainable eating behavior. The total SHEB score, designated as the primary outcome, increased progressively across MDA categories. Importantly, this association was also evident when MDA was modeled as a continuous variable, with each one-point increase in MDA score corresponding to an approximately 4.1-point higher total SHEB score after covariate adjustment. The consistency of the findings across categorical and continuous specifications supports the robustness of the observed cross-sectional association while reducing concerns related to information loss from categorization. After controlling the FDR across individual SHEB subdimensions, significant differences remained for use of quality labels, seasonal foods and food waste avoidance, local food, reduced meat consumption, and animal welfare. However, the animal-welfare association warrants particular caution because this subdimension showed very low internal consistency in the present sample (Cronbach’s α = 0.304), which may limit the reliability and interpretability of this finding. Among the more reliably measured subdimensions, the largest effect size was observed for reduced meat consumption. These findings suggest substantial alignment between Mediterranean diet adherence and several dimensions of sustainable eating; however, they should not necessarily be interpreted as evidence of independent behavioral constructs. Several components of the Mediterranean dietary pattern, including reduced meat consumption and preference for seasonal and locally sourced foods, conceptually overlap with dimensions captured by the SHEB Scale. Thus, part of the observed association may reflect shared construct content and common self-report methodology. However, the finding for local food appeared less robust than those for the other sustainability-related subdimensions: although the overall FDR-corrected association was significant, no individual pairwise comparison remained significant after Bonferroni adjustment. Therefore, this finding should be interpreted cautiously as evidence of an overall cross-sectional pattern rather than a clear difference between specific MDA adherence categories. Overall, these findings suggest alignment between MDA and sustainable eating behaviors, although some conceptual overlap exists between the two constructs. Several behaviors captured by SHEB, including reduced meat consumption and seasonal/local food choices, conceptually overlap with characteristics of a Mediterranean dietary pattern. Thus, the observed associations may partly reflect shared behavioral content rather than wholly independent relationships. Moreover, as both constructs were assessed using self-reported measures within the same assessment, common-method variance may have contributed to the observed associations. Accordingly, these findings are best interpreted as cross-sectional convergence between related dietary and sustainability-oriented behavioral constructs rather than evidence that Mediterranean diet adherence independently determines sustainable eating behavior.

The etiology of obesity is multifaceted, extending beyond energy imbalance to include behavioral, nutritional, and physiological factors that interact in complex ways [41]. Growing evidence suggests that sustainable and health-oriented eating behaviors may play a protective role against obesity by promoting balanced, plant-forward, and environmentally conscious food choices [9]. Such diets are typically rich in fiber, antioxidants, and phytochemicals, which enhance satiety, facilitate caloric reduction, modulate the gut microbiota, and reduce systemic inflammation, thereby improving metabolic health in both healthy and metabolically impaired individuals [12]. Supporting this evidence, analysis of data from the National Health and Nutrition Examination Survey (NHANES) 2007–2018 (n = 25,262) demonstrated that higher Sustainable Diet Index for the United States (SDI-US) scores—calculated from dietary intake, food expenditures, environmental impacts, and food practices—were associated with significantly lower odds of obesity (OR = 0.68, 95% CI 0.58–0.79; *p* < 0.001) [13]. Similarly, in a study of 210 adults in Türkiye (equally divided between white- and blue-collar employees), higher Sustainable Eating Attitude scores—reflecting greater importance placed on sustainably produced foods and willingness to pay more for them—were inversely associated with body weight, BMI, waist circumference, and percent body fat (*p* < 0.05 to *p* < 0.001) [14]. With respect to anthropometric outcomes, WHR was the only measure that remained significantly associated with SHEB tertiles after correction for multiple testing. Participants in the highest SHEB tertile had a lower adjusted WHR than those in the lowest tertile, with a moderate effect size. Although neck circumference also differed at the nominal level, this association did not withstand FDR correction and should therefore not be interpreted as a robust finding. Likewise, no FDR-corrected differences were observed for body weight, BMI, waist or hip circumference, or body composition measures. These results provide limited but potentially relevant evidence of a cross-sectional association between sustainable eating behavior and central fat distribution, specifically WHR, rather than evidence of a broader favorable anthropometric profile. Notably, these associations were not observed for BMI or body fat percentage, suggesting that sustainable eating behaviors may be more closely related to fat distribution than to overall adiposity in this sample. This pattern is consistent with evidence that central adiposity measures provide information on metabolic risk beyond general adiposity measures such as BMI [42,43]. Nevertheless, given the cross-sectional design and the absence of consistent associations across adiposity measures, these findings should be considered exploratory rather than evidence of a preferential effect on central adiposity.

A significant practice gap persists in empowering individuals with T2DM to transform dietary advice into sustainable eating behaviors [17]. Previous intervention studies suggest that practical cooking and food skills may facilitate diabetes self-management and sustainable dietary practices [16,18]. For instance, a culinary medicine intervention that combined practical cooking instruction with nutrition education led to a significant reduction in HbA1c levels among adults with T2DM who also received food prescriptions [19]. Participants with macrovascular complications had lower food skills and CSFS total scores at the nominal level; however, neither association remained statistically significant after FDR correction (both q = 0.056). These findings therefore represent exploratory signals rather than robust evidence of differences in cooking and food skills according to macrovascular complication status. Although previous intervention studies suggest that culinary and food skills training may support diabetes self-management, the present cross-sectional findings cannot establish whether lower food skills precede vascular complications or reflect behavioral changes following their diagnosis. Intervention studies specifically designed to evaluate cardiovascular outcomes are needed before clinical implications can be inferred.

The cardioprotective effects of the Mediterranean diet are primarily attributed to its unique nutrient composition and bioactive compounds. Rich in olive oil, fish, nuts, whole grains, fruits, and vegetables, the diet provides an abundance of monounsaturated and omega-3 fatty acids, polyphenols, antioxidants, and dietary fiber. These components have been linked to biological pathways involving oxidative stress, inflammation, endothelial function, lipid metabolism, and blood pressure regulation [4]. Consistent with this broader evidence, a longitudinal cohort study from Iran involving 22,187 adults with diabetes reported that greater adherence to the Mediterranean diet was associated with a significantly lower risk of cardiovascular disease among individuals with type 2 diabetes (OR = 0.61, 95% CI: 0.57–0.89; *p* < 0.001) [15]. In the present study, MDA scores were lower among participants with macrovascular complications at the nominal level (*p* = 0.018); however, this difference did not remain significant after FDR correction (q = 0.056). Accordingly, the present data should not be interpreted as providing independent evidence of an association between Mediterranean diet adherence and prevalent macrovascular complications. The direction of the observed difference is nevertheless compatible with findings from prospective studies linking greater Mediterranean diet adherence with lower cardiovascular risk, and warrants further investigation in adequately powered longitudinal studies.

Among the dietary variables examined, adjusted total protein intake was the only between-group difference that remained statistically significant after FDR correction. Importantly, however, the direction of this difference reversed after covariate and total-energy adjustment: crude protein intake was numerically higher among participants with macrovascular complications, whereas adjusted protein intake was lower in this group. This reversal indicates that the estimated between-group difference was strongly influenced by the covariate structure, including adjustment for total energy intake. Accordingly, the finding should be interpreted specifically as an association with adjusted protein intake rather than as evidence that participants with macrovascular complications consumed less protein in absolute, unadjusted terms. In considering the potential relevance of this adjusted association, previous evidence suggests that adequate protein intake, particularly from plant and lean animal sources, may support weight management, metabolic control, and functional health in individuals with diabetes [22]. Nevertheless, evidence linking protein intake specifically to cardiovascular outcomes remains less conclusive. A dose–response meta-analysis of prospective cohort studies reported a modest inverse association between total protein intake and all-cause mortality, whereas neither total nor animal protein intake was significantly associated with cardiovascular disease mortality [21]. Moreover, plant protein intake did not differ according to macrovascular complication status in the present study. Thus, although the observed difference in total protein intake remained robust to correction for multiple testing, its clinical and etiological significance requires confirmation in prospective studies.

Several other dietary variables, including total fat, saturated fatty acids, monounsaturated fatty acids, and dietary total antioxidant capacity, showed between-group differences at the nominal significance level but did not remain significant after FDR correction. Moreover, the nominal difference in dietary total antioxidant capacity was not reproduced in the sensitivity analysis using natural-log-transformed values to address marked residual non-normality. Accordingly, these findings should be interpreted as exploratory rather than confirmatory. This distinction is important because existing evidence supports a role for dietary fat quality in cardiovascular health, including benefits associated with reducing saturated fat intake and replacing it with unsaturated fatty acids [20,23], while higher dietary antioxidant exposure has been associated with more favorable cardiometabolic outcomes in observational studies [20,23,24,25]. However, the present data do not provide sufficiently robust evidence to extend these associations to prevalent macrovascular complications in this sample. Dietary intake was assessed using a single 24-h recall, which may not fully capture usual intake because of day-to-day variability. In addition, the relatively low reported energy intake in this predominantly obese sample suggests possible dietary under-reporting, which may have influenced dietary estimates and between-group comparisons. As energy misreporting was not formally assessed, the magnitude and direction of this potential bias cannot be determined. Reverse causation also remains plausible, as participants with established cardiovascular disease may have modified their dietary practices in response to diagnosis, medical advice, or treatment. Accordingly, the observed dietary differences should be interpreted as cross-sectional associations and hypothesis-generating signals rather than as evidence that these dietary characteristics preceded, contributed to, or protected against the development of macrovascular complications.

This study has several notable strengths. First, it integrates diet quality, sustainability-related eating behaviors, cooking and food skills, anthropometric and body composition measures, and clinical characteristics within a single analytical framework among adults with T2DM, providing a multidimensional perspective that remains relatively underexplored in this population. Second, the use of validated and culturally adapted instruments—including the Sustainable and Healthy Eating Behaviors (SHEB) Scale, Cooking Skills and Food Skills (CSFS) Scale, Mediterranean Diet Adherence (MDA) Scale, and IPAQ—together with standardized anthropometric assessments, supports the methodological consistency and interpretability of the findings. Third, the use of covariate-adjusted ANCOVA models enabled adjustment for several prespecified sociodemographic, clinical, and lifestyle factors, with additional adjustment for total energy intake in analyses of dietary variables. Importantly, applying the Benjamini–Hochberg FDR correction across conceptually defined families of exploratory outcomes further strengthened the analytical approach by reducing the likelihood that isolated findings were attributable to multiple testing. Another strength is the detailed assessment of anthropometric and body composition measures beyond BMI, including indices of central adiposity such as the waist-to-hip ratio and neck circumference, thereby allowing a broader characterization of the cardiometabolic phenotype. Finally, the simultaneous examination of sustainable eating behaviors, cooking and food skills, dietary intake, and macrovascular complication status provides a clinically relevant framework for identifying associations that warrant further investigation in adequately powered prospective and intervention studies.

The relatively small sample size and markedly unequal distribution across MDA categories, particularly the high-MDA group (n = 14), may have reduced the precision and stability of category-specific adjusted estimates and increased their sensitivity to model specification. Accordingly, these subgroup estimates should be interpreted cautiously. This concern was partly addressed by a complementary analysis that modeled MDA as a continuous variable, yielding findings consistent with the categorical analysis while avoiding information loss from categorization. Furthermore, the original sample-size calculation was conducted for the broader parent project and was not specifically based on the primary outcome, subgroup comparisons, or adjusted models examined in the present analysis. Therefore, the present analyses should not be interpreted as having been prospectively powered for all reported comparisons, particularly subgroup-based and extensively adjusted analyses. Although the FDR correction was applied to conceptually defined families of exploratory outcomes to address multiplicity, several nominal associations did not remain statistically significant after correction. These findings should therefore be considered hypothesis-generating and require confirmation in larger, prospectively designed studies.

The cross-sectional design precludes establishing temporality or causality; reverse causation is particularly relevant in analyses of macrovascular complications, as participants may have modified their dietary practices and lifestyle behaviors following a cardiovascular diagnosis or clinical management. Residual confounding may also remain despite covariate adjustment. Although sensitivity analyses additionally accounting for available major vascular risk factors—including smoking, hypertension, HbA1c, LDL cholesterol, and serum creatinine—generally supported the principal protein finding, unmeasured or incompletely characterized cardiovascular risk factors and medication-related influences cannot be excluded. In addition, although myocardial infarction and stroke histories were separately identifiable, the individual components of the macrovascular complication composite were not systematically coded as separate diagnostic variables. Therefore, the complete distribution of coronary artery disease, peripheral arterial disease, heart failure, and overlap between individual conditions could not be reliably reconstructed retrospectively. Furthermore, dietary intake, physical activity, and behavioral characteristics were largely assessed using self-reported measures and are therefore susceptible to recall and social desirability biases. Although internal consistency was good for the overall SHEB scale (α = 0.858), reliability varied across its subdimensions, with particularly low internal consistency for animal welfare (α = 0.304) and lower estimates for seasonal food and avoiding food waste (α = 0.597) and low-fat foods (α = 0.615). Accordingly, findings involving these subdimensions should be interpreted with caution. The use of a single 24-h dietary recall may not adequately represent usual dietary intake or account for day-to-day variation, while the relatively low reported energy intake raises the possibility of dietary under-reporting, which was not formally assessed. Finally, the single-center design and predominance of female participants may limit the generalizability of the findings to broader populations with T2DM.

## 5. Conclusions

In adults with T2DM, greater Mediterranean diet adherence was cross-sectionally associated with higher overall sustainable and healthy eating behavior scores and with several sustainability-related behavioral dimensions after correction for multiple testing. Among anthropometric outcomes, higher SHEB scores were associated with a lower waist-to-hip ratio, whereas other anthropometric and body composition measures did not remain significant after FDR correction. In analyses according to macrovascular complication status, lower adjusted protein intake was the only difference that remained statistically significant after multiplicity correction, although the reversal in direction between crude and adjusted estimates warrants cautious interpretation; nominal differences in food skills, Mediterranean diet adherence, fat intake, and dietary antioxidant capacity should therefore be considered exploratory. Given the cross-sectional design, limited subgroup sizes, and reliance on a single 24-h dietary recall, these findings do not establish temporal or causal relationships. Larger prospective studies are needed to determine whether adherence to the Mediterranean diet, sustainable eating behaviors, and cooking and food skills are associated with subsequent cardiometabolic and vascular outcomes and to provide a basis for appropriately designed intervention studies.

## Figures and Tables

**Table 1:** Baseline sociodemographic, clinical, and lifestyle characteristics of adults with type 2 diabetes mellitus (T2DM).

Age (years)	58.6 ± 10.0 (60.0)
Duration of T2DM (years)	11.6 ± 6.5 (10.5)
Sex, n (%)	
Men	19 (15.8%)
Women	101 (84.2%)
Current smoker, n (%)	18 (15.0%)
Physical activity level (MET-min/week)	418.1 ± 547.1 (219.2)
Educational level	
Primary education or below, n (%)	74 (61.7%)
Above primary education, n (%)	46 (38.3%)
Marital status, n (%)	
Married	99 (82.5%)
Single	21 (17.5%)
Income level	
Financial insufficiency	29 (24.2%)
Financial adequacy	76 (63.3%)
Financial surplus	15 (12.5%)
Body mass index (kg/m^2^)	32.9 ± 6.1 (32.0)
<30 kg/m^2^, n (%)	43 (35.8%)
≥30 kg/m^2^, n (%)	77 (64.2%)
Macrovascular complications	
Absent, n (%)	82 (68.3%)
≥1 present, n (%)	38 (31.7%)
Oral antidiabetic therapy, n (%)	112 (93.3%)
Insulin therapy, n (%)	55 (45.8%)
HbA1c (%)	7.8 ± 1.7 (7.4)
SHEB total score	131.9 ± 20.2 (131.0)
CSFS total score	161.1 ± 35.2 (163.5)
MDA score	6.44 ± 2.41 (6.0)

SHEB: Sustainable and healthy eating behaviors; CSFS: Cooking skills and food skills; MDA: Mediterranean diet adherence. Data are presented as mean ± standard deviation with the median (in parentheses) for continuous variables, and as number (percentage) for categorical variables.

**Table 2:** Crude and adjusted means of Sustainable and Healthy Eating Behaviors (SHEB) subdimensions and total scores across Mediterranean Diet Adherence categories among adults with T2DM.

	Mediterranean Diet Adherence						
	Low (n = 52)	Moderate (n = 54)	High (n = 14)	F_adj_	*p*-Value	FDR q-Value	Partial η^2^
**Quality labels (regional and organic products)**							
Crude Mean ± SD	28.37 ± 6.83	31.81 ± 6.22	34.14 ± 4.61				
Adjusted Mean ± SE	28.19 ± 0.89 ^a^	31.99 ± 0.88 ^b^	34.13 ± 1.70 ^b^	6.923	0.001	0.002	0.113
**Seasonal foods and food waste avoidance**							
Crude Mean ± SD	34.83 ± 6.18	36.70 ± 3.66	38.50 ± 4.36				
Adjusted Mean ± SE	34.62 ± 0.70 ^a^	36.83 ± 0.70 ^a^	38.77 ± 1.33 ^b^	4.740	0.011	0.019	0.080
**Healthy and balanced eating**							
Crude Mean ± SD	18.42 ± 3.90	18.96 ± 4.01	18.93 ± 4.16				
Adjusted Mean ± SE	18.43 ± 0.58	18.92 ± 0.57	19.06 ± 1.10	0.230	0.795	0.795	0.004
**Local food**							
Crude Mean ± SD	7.29 ± 3.83	9.20 ± 4.59	10.21 ± 4.95				
Adjusted Mean ± SE	7.23 ± 0.61	9.25 ± 0.60	10.25 ± 1.15	4.056	0.020	0.028	0.069
**Reduced meat consumption**							
Crude Mean ± SD	7.67 ± 3.43	10.09 ± 4.17	11.71 ± 3.38				
Adjusted Mean ± SE	7.61 ± 0.54 ^a^	10.15 ± 0.54 ^b^	11.72 ± 1.04 ^b^	8.570	<0.001	0.001	0.136
**Animal welfare**							
Crude Mean ± SD	12.48 ± 3.72	15.11 ± 3.54	16.86 ± 3.35				
Adjusted Mean ± SE	12.51 ± 0.52 ^a^	15.05 ± 0.51 ^b^	17.01 ± 0.99 ^b^	10.454	<0.001	<0.001	0.161
**Low-fat choices**							
Crude Mean ± SD	14.13 ± 3.09	14.70 ± 2.42	15.43 ± 2.10				
Adjusted Mean ± SE	14.09 ± 0.38	14.71 ± 0.38	15.58 ± 0.73	1.800	0.170	0.198	0.032
**Total score**							
Crude Mean ± SD	123.2 ± 19.6	136.6 ± 18.0	145.8 ± 16.9				
Adjusted Mean ± SE	122.7 ± 2.7 ^a^	136.9 ± 2.6 ^b^	146.5 ± 5.1 ^b^	11.537	<0.001	-	0.175

Values are presented as mean ± standard deviation (crude) and adjusted mean ± standard error (SE). Fadj represents the F statistic from the covariate-adjusted ANCOVA model, and *p*-values represent the corresponding nominal significance levels (unadjusted for multiple testing). ANCOVA models were adjusted for age, duration of type 2 diabetes mellitus, sex, educational level, marital status, antidiabetic medication use, insulin therapy, and total physical activity level. FDR-adjusted q-values for the seven exploratory SHEB subdimensions were calculated using the Benjamini–Hochberg procedure, with q < 0.05 considered statistically significant after correction for multiple testing. The total SHEB score was designated as the primary outcome and was therefore not included in the FDR correction. ^a^, ^b^: Different superscript letters indicate statistically significant Bonferroni-adjusted pairwise differences (*p* < 0.05). For local food, the overall ANCOVA remained significant after FDR correction, but none of the pairwise comparisons remained significant after Bonferroni adjustment; therefore, superscript letters are not shown. Partial η^2^ indicates effect size.

**Table 3:** Crude and adjusted means of anthropometric and body composition measures across tertiles of Sustainable and Healthy Eating Behaviors total score among adults with T2DM.

	Sustainable and Healthy Eating Behaviors Total Score						
	<124 (n = 40)	124–140 (n = 40)	>140 (n = 40)	F_adj_	*p*-Value	FDR q-Value	Partial η^2^
**Body weight (kg)**							
Crude Mean ± SD	83.40 ± 19.11	81.94 ± 12.73	80.13 ± 15.81				
Adjusted Mean ± SE	83.67 ± 2.51	81.32 ± 2.54	80.49 ± 2.50	0.433	0.650	0.918	0.008
**Body mass index (kg/m^2^)**							
Crude Mean ± SD	33.48 ± 7.01	33.60 ± 4.96	31.56 ± 6.08				
Adjusted Mean ± SE	33.34 ± 0.92	33.45 ± 0.93	31.86 ± 0.92	0.931	0.397	0.662	0.017
**Waist circumference (cm)**							
Crude Mean ± SD	109.2 ± 13.5	108.3 ± 10.8	103.2 ± 12.9				
Adjusted Mean ± SE	109.0 ± 2.0	108.5 ± 2.0	103.2 ± 2.0	2.664	0.074	0.247	0.047
**Hip circumference (cm)**							
Crude Mean ± SD	115.5 ± 12.5	116.0 ± 11.3	114.1 ± 11.1				
Adjusted Mean ± SE	115.3 ± 1.8	115.9 ± 1.9	114.4 ± 1.8	0.161	0.852	0.944	0.003
**Waist-to-hip ratio**							
Crude Mean ± SD	0.95 ± 0.05	0.94 ± 0.06	0.90 ± 0.06				
Adjusted Mean ± SE	0.95 ± 0.01 ^a^	0.94 ± 0.01 ^a^	0.90 ± 0.01 ^b^	7.479	0.001	0.010	0.121
**Neck circumference (cm)**							
Crude Mean ± SD	39.18 ± 3.39	38.30 ± 3.07	37.55 ± 3.37				
Adjusted Mean ± SE	39.32 ± 0.52	38.21 ± 0.52	37.50 ± 0.52	3.163	0.046	0.230	0.055
**Body fat percentage (%)**							
Crude Mean ± SD	38.03 ± 7.88	38.83 ± 6.10	37.73 ± 9.22				
Adjusted Mean ± SE	37.61 ± 0.93	38.63 ± 0.95	38.35 ± 0.93	0.310	0.734	0.918	0.006
**Fat mass (kg)**							
Crude Mean ± SD	32.83 ± 12.74	32.13 ± 8.76	31.82 ± 12.58				
Adjusted Mean ± SE	32.64 ± 1.72	31.80 ± 1.75	32.34 ± 1.72	0.057	0.944	0.944	0.001
**Fat-free mass (kg)**							
Crude Mean ± SD	50.61 ± 8.63	49.70 ± 7.36	48.65 ± 9.24				
Adjusted Mean ± SE	51.06 ± 1.12	49.40 ± 1.13	48.51 ± 1.11	1.347	0.264	0.660	0.024
**Total body water (%)**							
Crude Mean ± SD	37.07 ± 6.29	36.22 ± 5.68	36.31 ± 6.26				
Adjusted Mean ± SE	37.42 ± 0.73	36.12 ± 0.75	36.06 ± 0.74	1.076	0.345	0.662	0.019

Values are presented as mean ± standard deviation (crude) and adjusted mean ± standard error (SE). Fadj represents the F statistic from the covariate-adjusted ANCOVA model, and *p*-values represent the corresponding nominal significance levels (unadjusted for multiple testing). ANCOVA models were adjusted for age, duration of type 2 diabetes mellitus, sex, educational level, marital status, antidiabetic medication use, insulin therapy, and total physical activity level. *p*-values for the 10 exploratory anthropometric and body-composition outcomes were adjusted for multiple testing using the Benjamini–Hochberg false discovery rate (FDR) procedure; FDR-adjusted q-values < 0.05 were considered statistically significant after correction for multiple testing. Pairwise post-hoc comparisons were interpreted only for outcomes that remained significant after FDR correction; different superscript letters (^a^, ^b^) indicate statistically significant Bonferroni-adjusted pairwise differences (*p* < 0.05). Partial η^2^ indicates effect size.

**Table 4:** Crude and adjusted means of cooking skills score, food skills score, Cooking Skills and Food Skills total score, Sustainable and Healthy Eating Behaviors total score, Mediterranean Diet Adherence score, and dietary components according to macrovascular complication status among adults with T2DM.

	Macrovascular Complications					
	Absent (n = 82)	≥1 Present (n = 38)	F_adj_	*p*-Value	FDR q-Value	Partial η^2^
**Cooking skills score**						
Crude Mean ± SD	78.54 ± 17.31	70.58 ± 16.10				
Adjusted Mean ± SE	77.39 ± 1.41	73.06 ± 2.15	2.586	0.111	0.197	0.023
**Food skills score**						
Crude Mean ± SD	89.01 ± 19.42	76.55 ± 22.73				
Adjusted Mean ± SE	88.35 ± 2.07	78.00 ± 3.17	6.814	0.010	0.056	0.058
**CSFS total score**						
Crude Mean ± SD	167.5 ± 34.0	147.1 ± 33.9				
Adjusted Mean ± SE	165.7 ± 3.0	151.0 ± 4.6	6.447	0.013	0.056	0.055
**SHEB total score**						
Crude Mean ± SD	131.1 ± 21.9	133.5 ± 15.9				
Adjusted Mean ± SE	130.5 ± 2.3	134.9 ± 3.6	0.935	0.336	0.448	0.009
**MDA score**						
Crude Mean ± SD	6.76 ± 2.50	5.76 ± 2.10				
Adjusted Mean ± SE	6.84 ± 0.27	5.59 ± 0.42	5.799	0.018	0.056	0.050
**Carbohydrate (g/day)**						
Crude Mean ± SD	120.2 ± 54.5	145.5 ± 45.5				
Adjusted Mean ± SE	129.6 ± 3.5	125.2 ± 5.4	0.419	0.519	0.639	0.004
**Dietary fiber (g/day)**						
Crude Mean ± SD	13.21 ± 6.07	17.94 ± 8.26				
Adjusted Mean ± SE	14.60 ± 0.61	14.94 ± 0.95	0.084	0.773	0.773	0.001
**Protein (g/day)**						
Crude Mean ± SD	47.42 ± 18.31	51.12 ± 24.39				
Adjusted Mean ± SE	51.37 ± 1.39	42.60 ± 2.15	10.482	0.002	0.032	0.088
**Plant protein (g/day)**						
Crude Mean ± SD	19.18 ± 10.18	25.64 ± 11.40				
Adjusted Mean ± SE	20.99 ± 0.86	21.75 ± 1.34	0.203	0.653	0.715	0.002
**Total fat (g/day)**						
Crude Mean ± SD	40.81 ± 19.23	57.92 ± 30.43				
Adjusted Mean ± SE	44.42 ± 1.44	50.12 ± 2.24	4.059	0.046	0.092	0.036
**Saturated fatty acids (g/day)**						
Crude Mean ± SD	15.31 ± 7.91	23.16 ± 14.27				
Adjusted Mean ± SE	16.64 ± 0.78	20.30 ± 1.20	5.791	0.018	0.056	0.050
**Monounsaturated fatty acids (g/day)**						
Crude Mean ± SD	14.11 ± 7.47	21.04 ± 11.48				
Adjusted Mean ± SE	15.35 ± 0.66	18.35 ± 1.02	5.463	0.021	0.056	0.048
**Polyunsaturated fatty acids (g/day)**						
Crude Mean ± SD	7.72 ± 5.31	9.18 ± 5.58				
Adjusted Mean ± SE	8.50 ± 0.47	7.49 ± 0.74	1.207	0.274	0.399	0.011
**Cholesterol (mg/day)**						
Crude Mean ± SD	248.1 ± 148.1	285.8 ± 189.6				
Adjusted Mean ± SE	264.3 ± 16.0	251.0 ± 24.8	0.182	0.670	0.715	0.002
**ORAC (μmol TE/day)**						
Crude Mean ± SD	2298.3 ± 2298.9	3567.1 ± 2364.1				
Adjusted Mean ± SE	2483.4 ± 262.2	3167.8 ± 406.6	1.778	0.185	0.296	0.016
**Total antioxidant capacity (mmol/day)**						
Crude Mean ± SD	5.21 ± 13.08	4.26 ± 2.96				
Adjusted Mean ± SE	6.55 ± 1.17	1.37 ± 1.82	5.100	0.026	0.059	0.045

SHEB: Sustainable and Healthy Eating Behaviors; CSFS: Cooking Skills and Food Skills; MDA: Mediterranean Diet Adherence. ORAC: Oxygen radical absorbance capacity; TE: Trolox equivalents. Values are presented as mean ± standard deviation (crude) and adjusted mean ± standard error (SE). Fadj represents the F statistic from the covariate-adjusted ANCOVA model, and *p*-values represent the corresponding nominal (unadjusted for multiple testing) significance levels. ANCOVA models were adjusted for age, duration of type 2 diabetes mellitus, sex, educational level, marital status, antidiabetic medication use, insulin therapy, and total physical activity level; dietary component comparisons were additionally adjusted for total energy intake. FDR-adjusted q-values were calculated using the Benjamini–Hochberg procedure across the 16 exploratory outcomes examined according to macrovascular complication status, with q < 0.05 considered statistically significant after correction for multiple testing. Partial η^2^ indicates effect size.

## Data Availability

The data presented in this study are not publicly available due to ethical and privacy restrictions related to participant confidentiality and the conditions of informed consent approved by the institutional ethics committee. Anonymized data may be made available by the corresponding author upon reasonable request and, where applicable, subject to approval by the relevant ethics committee.

## Supplementary Materials

The following supporting information can be downloaded at https://www.mdpi.com/article/10.3390/healthcare14182934/s1, Table S1. Diagnostic assessment of ANCOVA assumptions and transformation sensitivity analyses for selected dietary outcomes. Table S2. Expanded vascular-risk-adjusted sensitivity analyses of dietary variables according to macrovascular complication status.

## References

[1] American Diabetes Association Professional Practice Committee (2025). 10. Cardiovascular Disease and Risk Management: Standards of Care in Diabetes-2025. Diabetes Care.

[2] Willett W., Rockström J., Loken B., Springmann M., Lang T., Vermeulen S., Garnett T., Tilman D., DeClerck F., Wood A. (2019). Food in the anthropocene: The EAT-lancet commission on healthy diets from sustainable food systems. Lancet.

[3] Franchini C., Biasini B., Sogari G., Wongprawmas R., Andreani G., Dolgopolova I., Gómez M.I., Roosen J., Menozzi D., Mora C. (2024). Adherence to the Mediterranean Diet and its association with sustainable dietary behaviors, sociodemographic factors, and lifestyle: A cross-sectional study in US University students. Nutr. J..

[4] Scaglione S., Di Chiara T., Daidone M., Tuttolomondo A. (2025). Effects of the Mediterranean Diet on the Components of Metabolic Syndrome Concerning the Cardiometabolic Risk. Nutrients.

[5] Metin Z.E., Celik O.M., Koc N. (2024). Relationship between adherence to the Mediterranean diet, sustainable and healthy eating behaviors, and climate change awareness: A cross-sectional study from Turkey. Nutrition.

[6] Papamichou D., Panagiotakos D.B., Itsiopoulos C. (2019). Dietary patterns and management of type 2 diabetes: A systematic review of randomised clinical trials. Nutr. Metab. Cardiovasc. Dis..

[7] Salvia M.G., Quatromoni P.A. (2023). Behavioral approaches to nutrition and eating patterns for managing type 2 diabetes: A review. Am. J. Med. Open.

[8] Umphonsathien M., Rattanasian P., Lokattachariya S., Suansawang W., Boonyasuppayakorn K., Khovidhunkit W. (2022). Effects of intermittent very-low calorie diet on glycemic control and cardiovascular risk factors in obese patients with type 2 diabetes mellitus: A randomized controlled trial. J. Diabetes Investig..

[9] Mambrini S.P., Penzavecchia C., Menichetti F., Foppiani A., Leone A., Pellizzari M., Sileo F., Battezzati A., Bertoli S., De Amicis R. (2025). Plant-based and sustainable diet: A systematic review of its impact on obesity. Obes. Rev..

[10] Weller S.C., Baer R., Nash A., Perez N. (2017). Discovering successful strategies for diabetic self-management: A qualitative comparative study. BMJ Open Diabetes Res. Care.

[11] Weller S.C., Vickers B.N. (2021). Identifying sustainable lifestyle strategies for maintaining good glycemic control: A validation of qualitative findings. BMJ Open Diabetes Res. Care.

[12] Leziak A., Lipina J., Reclik M., Kocelak P. (2025). Dietary Modulation of Metabolic Health: From Bioactive Compounds to Personalized Nutrition. Metabolites.

[13] Jung S., Young H.A., Simmens S.J., Braffett B.H., Ogden C.L. (2023). The cross-sectional association between a sustainable diet index and obesity among US adults. Obesity.

[14] Şahin Bayram S., Kızıltan G. (2024). The Correlation between Knowledge of Food Sustainability, Sustainable Eating Attitudes, and Adherence to the Mediterranean Diet among Blue- and White-Collar Employees. Sustainability.

[15] Ghaemi F., Firouzabadi F.D., Moosaie F., Shadnoush M., Poopak A., Kermanchi J., Abhari S.M.F., Forouzanfar R., Mansournia M.A., Khosravi A. (2021). Effects of a Mediterranean diet on the development of diabetic complications: A longitudinal study from the nationwide diabetes report of the National Program for Prevention and Control of Diabetes (NPPCD 2016–2020). Maturitas.

[16] Biasini B., Rosi A., Giopp F., Turgut R., Scazzina F., Menozzi D. (2021). Understanding, promoting and predicting sustainable diets: A systematic review. Trends Food Sci. Technol..

[17] Fredericks L., Thomas O., Imamura A., MacLaren J., McClure A., Khalil J., Massa J. (2025). Will a Programmatic Framework Integrating Food Is Medicine Achieve Value on Investment?. J. Gen. Intern. Med..

[18] Heredia N.I., Macias-Navarro L., Guevara D.C., Sharma S.V., Chow J., Bentley S.S., Chukuigwe O., Pappa A., McWhorter J.W. (2025). Testing of a Culinary Medicine Intervention for Racially/Ethnically Diverse Adults With Type 2 Diabetes. J. Nutr. Educ. Behav..

[19] Sharma S.V., McWhorter J.W., Chow J., Danho M.P., Weston S.R., Chavez F., Moore L.S., Almohamad M., Gonzalez J., Liew E. (2021). Impact of a Virtual Culinary Medicine Curriculum on Biometric Outcomes, Dietary Habits, and Related Psychosocial Factors among Patients with Diabetes Participating in a Food Prescription Program. Nutrients.

[20] Hooper L., Martin N., Jimoh O.F., Kirk C., Foster E., Abdelhamid A.S. (2020). Reduction in saturated fat intake for cardiovascular disease. Cochrane Database Syst. Rev..

[21] Naghshi S., Sadeghi O., Willett W.C., Esmaillzadeh A. (2020). Dietary intake of total, animal, and plant proteins and risk of all-cause, cardiovascular, and cancer mortality: Systematic review and dose-response meta-analysis of prospective cohort studies. BMJ.

[22] Pfeiffer A.F.H., Pedersen E., Schwab U., Riserus U., Aas A.M., Uusitupa M., Thanopoulou A., Kendall C., Sievenpiper J.L., Kahleová H. (2020). The Effects of Different Quantities and Qualities of Protein Intake in People with Diabetes Mellitus. Nutrients.

[23] Schwingshackl L., Zahringer J., Beyerbach J., Werner S.S., Heseker H., Koletzko B., Meerpohl J.J. (2021). Total Dietary Fat Intake, Fat Quality, and Health Outcomes: A Scoping Review of Systematic Reviews of Prospective Studies. Ann. Nutr. Metab..

[24] Zhang J., Chen Y., Zou L., Jin L., Yang B., Shu Y., Gong R. (2023). Dose-response relationship between dietary antioxidant intake and diabetic kidney disease in the US adults with diabetes. Acta Diabetol..

[25] Zujko M.E., Waskiewicz A., Witkowska A.M., Cicha-Mikolajczyk A., Zujko K., Drygas W. (2022). Dietary Total Antioxidant Capacity-A New Indicator of Healthy Diet Quality in Cardiovascular Diseases: A Polish Cross-Sectional Study. Nutrients.

[26] Zakowska-Biemans S., Pieniak Z., Kostyra E., Gutkowska K. (2019). Searching for a Measure Integrating Sustainable and Healthy Eating Behaviors. Nutrients.

[27] Koksal E., Bilici S., Citar Daziroglu M.E., Erdogan Govez N. (2023). Validity and Reliability of the Turkish Version of the Sustainable and Healthy Eating Behaviors Scale. Br. J. Nutr..

[28] Lavelle F., McGowan L., Hollywood L., Surgenor D., McCloat A., Mooney E., Caraher M., Raats M., Dean M. (2017). The development and validation of measures to assess cooking skills and food skills. Int. J. Behav. Nutr. Phys. Act..

[29] Keleş G., Akçil Ok M. (2021). Pişirme ve Yiyecek Hazırlama Becerileri Ölçeğinin Türkçe Geçerlik ve Güvenirliğinin İncelenmesi. J. Nutr. Diet..

[30] Martinez-Gonzalez M.A., Corella D., Salas-Salvado J., Ros E., Covas M.I., Fiol M., Wärnberg J., Arós F., Ruíz-Gutiérrez V., Lamuela-Raventós R.M. (2012). Cohort profile: Design and methods of the PREDIMED study. Int. J. Epidemiol..

[31] Bekar C., Goktas Z. (2023). Validation of the 14-item mediterranean diet adherence screener. Clin. Nutr. ESPEN.

[32] Ayoub J.J., Haidar S.A., Blaak E.E., De Vries N.K. (2025). Determinants of adherence to the Mediterranean diet among individuals with type 2 diabetes mellitus living in Mediterranean countries: A systematic review. Front. Nutr..

[33] García-Conesa M.T., Philippou E., Pafilas C., Massaro M., Quarta S., Andrade V., Jorge R., Chervenkov M., Ivanova T., Dimitrova D. (2020). Exploring the Validity of the 14-Item Mediterranean Diet Adherence Screener (MEDAS): A Cross-National Study in Seven European Countries around the Mediterranean Region. Nutrients.

[34] Rakıcıoğlu N., Tek N.A., Ayaz N., Pekcan G. (2009). Yemek ve Besin Fotoğraf Kataloğu.

[35] Craig C.L., Marshall A.L., Sjostrom M., Bauman A.E., Booth M.L., Ainsworth B.E., Pratt M., Ekelund U.L., Yngve A., Sallis J.F. (2003). International physical activity questionnaire: 12-country reliability and validity. Med. Sci. Sports Exerc..

[36] Saglam M., Arikan H., Savci S., Inal-Ince D., Bosnak-Guclu M., Karabulut E., Tokgozoglu L. (2010). International physical activity questionnaire: Reliability and validity of the Turkish version. Percept. Mot. Ski..

[37] World Health Organization (WHO) Physical Status: The Use of and Interpretation of Anthropometry: Report of a WHO Expert Committee. https://iris.who.int/handle/10665/37003.

[38] Czernichow S., Kengne A.P., Stamatakis E., Hamer M., Batty G.D. (2011). Body mass index, waist circumference and waist-hip ratio: Which is the better discriminator of cardiovascular disease mortality risk?: Evidence from an individual-participant meta-analysis of 82 864 participants from nine cohort studies. Obes. Rev..

[39] Sato R., Sawaya Y., Ishizaka M., Yin L., Shiba T., Hirose T., Urano T. (2024). Neck circumference is a highly reliable anthropometric measure in older adults requiring long-term care. PeerJ.

[40] Bôto J.M., Rocha A., Miguéis V., Meireles M., Neto B. (2022). Sustainability Dimensions of the Mediterranean Diet: A Systematic Review of the Indicators Used and Its Results. Adv. Nutr..

[41] Feraco A., Lombardo M., Armani A. (2025). The Effect of Lifestyle and Eating Habits on Obesity. Nutrients.

[42] Dardari Z., Yao Z., Zhang J., Bakoyannis G., Nan H., Staten L.K., Jha K.K., Tasdighi E., Jelwan Y., Burka S. (2026). Risk Reclassification Beyond BMI by Waist Circumference and Waist-to-Hip Ratio Across 9 Cardiovascular Outcomes: Results From the Cross-Cohort Collaboration. J. Am. Coll. Cardiol..

[43] Maleki S., Hosseinpanah F., Mahdavi M., Momenan A.A., Ebadi S.A., Rahmani F., Azizi F., Valizadeh M. (2026). Associations between indices of body composition and metabolic status in normal-weight adults: A cross-sectional study of the Tehran Lipid and Glucose Study. BMJ Open.

