# Supplementary material for: Mediterranean Diet Adherence and Sustainable Eating Behaviors in Type 2 Diabetes: A Cross-Sectional Analysis of Adiposity, Cooking and Food Skills, and Macrovascular Complications
Source: Healthcare (Basel). 2026 Sep 9;14(18):2934. doi: 10.3390/healthcare14182934 (PMC13606819; doi:10.3390/healthcare14182934)
Supplement: Supplementary file 1 [file healthcare-14-02934-s001.zip › healthcare-4524601-supplementary.pdf]

**Supplementary Table S1.** Diagnostic assessment of ANCOVA assumptions and transformation sensitivity analyses for selected dietary outcomes

**Panel A.** Diagnostic assessment of the ORAC ANCOVA model

| Diagnostic assessment | Result |
|---|---|
| Standardized residual skewness | 0.895 |
| Standardized residual kurtosis | 0.850 |
| Shapiro–Wilk p-value for standardized residuals | <0.001 |
| Standardized residual range | −1.57 to 3.47 |
| Studentized residual range | −1.69 to 3.58 |
| Maximum Cook's distance | 0.06 |
| Residual-versus-predicted pattern | No clear systematic or funnel-shaped pattern |
| Homogeneity of regression slopes | No significant group × continuous-covariate interactions* |
| VIF range | 1.041–1.270† |
| Tolerance range | 0.787–0.960† |
| Log-transformation assessment | Did not improve residual distribution |
| Macrovascular complication effect‡ | F=3.508; p=0.064; partial $\eta^2$=0.032 |

**Panel B.** Transformation sensitivity analysis for dietary total antioxidant capacity

| Diagnostic assessment | Original scale | Natural-log-transformed |
|---|---|---|
| Standardized residual skewness | 4.967 | −0.327 |
| Standardized residual kurtosis | 34.125 | 0.476 |
| Shapiro–Wilk p-value for standardized residuals | <0.001 | 0.495 |
| Standardized residual range | −1.65 to 7.36 | −2.93 to 2.61 |
| Studentized residual range | −1.82 to 7.85 | −3.10 to 2.79 |
| Maximum Cook's distance | 0.78 | 0.12 |
| Residual-distribution assessment | Marked non-normality | Substantially improved |
| Macrovascular complication effect | p=0.026; q=0.059 | F=0.387; p=0.535 |

**Values summarize ANCOVA diagnostic assessments and transformation sensitivity analyses for dietary outcomes requiring particular attention regarding model assumptions. Residual distributions were evaluated using standardized and studentized residuals, skewness, kurtosis, Shapiro–Wilk tests, and Q–Q plots. Residual-versus-predicted plots were visually inspected for evidence of heteroscedasticity, and Cook's distance was used to assess potentially influential observations. Homogeneity of regression slopes was assessed using macrovascular complication status × continuous-covariate interaction terms. Collinearity among continuous covariates was evaluated using variance inflation factors (VIFs) and tolerance values.***For the ORAC model, interactions between macrovascular complication status and age, diabetes duration, HbA1c, LDL cholesterol, serum creatinine, physical activity, and total energy intake were not statistically significant (all p≥0.272).
†VIF and tolerance values refer to the continuous covariates included in the expanded ORAC model; the observed ranges provided no evidence of problematic multicollinearity.
‡The ORAC estimate shown is from the expanded vascular-risk-adjusted sensitivity model.
**Abbreviations:** ORAC, oxygen radical absorbance capacity; VIF, variance inflation factor; q, false discovery rate-adjusted p-value.

**Supplementary Table S2.** Expanded vascular-risk-adjusted sensitivity analyses of dietary variables according to macrovascular complication status.

| Outcome | No macrovascular complication Adjusted mean ± SE | Macrovascular complication Adjusted mean ± SE | F | p-value | Partial η² |
|---|---|---|---|---|---|
| Total protein (g/day) | 54.85 ± 2.76 | 45.07 ± 3.07 | **13.326** | **<0.001** | **0.113** |
| Plant protein (g/day) | 23.21 ± 1.78 | 23.93 ± 1.98 | 0.174 | 0.678 | 0.002 |
| Total fat (g/day) | 39.13 ± 2.95 | 45.69 ± 3.28 | **5.252** | **0.024** | **0.048** |
| Saturated fatty acids (g/day) | 13.26 ± 1.56 | 17.42 ± 1.73 | **7.609** | **0.007** | **0.068** |
| Monounsaturated fatty acids (g/day) | 13.42 ± 1.32 | 16.84 ± 1.47 | **7.085** | **0.009** | **0.063** |
| Polyunsaturated fatty acids (g/day) | 8.49 ± 1.01 | 7.49 ± 1.12 | 1.047 | 0.309 | 0.010 |
| Dietary cholesterol (mg/day) | 212.69 ± 32.29 | 189.75 ± 35.87 | 0.537 | 0.465 | 0.005 |
| ORAC (µmol TE/day) | 1961.71 ± 543.28 | 2948.04 ± 603.50 | 3.508 | 0.064 | 0.032 |
| ln-transformed dietary total antioxidant capacity | 0.711 ± 0.238 | 0.650 ± 0.265 | 0.069 | 0.793 | 0.001 |

Values are presented as estimated marginal means ± standard error (SE). Sensitivity ANCOVA models were adjusted for smoking status, hypertension, HbA1c, LDL cholesterol, serum creatinine, age, diabetes duration, sex, oral antidiabetic medication use, insulin therapy, physical activity, and total energy intake. F statistics, p-values, and partial η² values refer to the main effect of macrovascular complication status.

These expanded models were performed as sensitivity analyses to evaluate potential residual vascular confounding and were not intended to replace the prespecified primary models. Reported p-values are nominal and should be interpreted as exploratory.

Dietary total antioxidant capacity was additionally examined after natural-log transformation because of residual non-normality in the original-scale model. **Abbreviations:** ORAC, oxygen radical absorbance capacity; SE, standard error.
